# A Novel Smartphone App for the Measurement of Ultra–Short-Term and Short-Term Heart Rate Variability: Validity and Reliability Study

**DOI:** 10.2196/18761

**Published:** 2020-07-31

**Authors:** Yung-Sheng Chen, Wan-An Lu, Jeffrey C Pagaduan, Cheng-Deng Kuo

**Affiliations:** 1 Department of Exercise and Health Sciences University of Taipei Taipei Taiwan; 2 Institute of Cultural Asset and Reinvention Fo-Guang University Yilan Taiwan; 3 College of Health and Medicine School of Health Sciences University of Tasmania Launceston Australia; 4 Department of Medical Research Taipei Veterans General Hospital Taipei Taiwan; 5 Tanyu Research Laboratory Taipei Taiwan

**Keywords:** heart rate variability, smartphone, reproducibility, limits of agreement, autonomic nervous function

## Abstract

**Background:**

Smartphone apps for heart rate variability (HRV) measurement have been extensively developed in the last decade. However, ultra–short-term HRV recordings taken by wearable devices have not been examined.

**Objective:**

The aims of this study were the following: (1) to compare the validity and reliability of ultra–short-term and short-term HRV time-domain and frequency-domain variables in a novel smartphone app, Pulse Express Pro (PEP), and (2) to determine the agreement of HRV assessments between an electrocardiogram (ECG) and PEP.

**Methods:**

In total, 60 healthy adults were recruited to participate in this study (mean age 22.3 years [SD 3.0 years], mean height 168.4 cm [SD 8.0 cm], mean body weight 64.2 kg [SD 11.5 kg]). A 5-minute resting HRV measurement was recorded via ECG and PEP in a sitting position. Standard deviation of normal R-R interval (SDNN), root mean square of successive R-R interval (RMSSD), proportion of NN50 divided by the total number of RR intervals (pNN50), normalized very-low–frequency power (nVLF), normalized low-frequency power (nLF), and normalized high-frequency power (nHF) were analyzed within 9 time segments of HRV recordings: 0-1 minute, 1-2 minutes, 2-3 minutes, 3-4 minutes, 4-5 minutes, 0-2 minutes, 0-3 minutes, 0-4 minutes, and 0-5 minutes (standard). Standardized differences (ES), intraclass correlation coefficients (ICC), and the Spearman product-moment correlation were used to compare the validity and reliability of each time segment to the standard measurement (0-5 minutes). Limits of agreement were assessed by using Bland-Altman plot analysis.

**Results:**

Compared to standard measures in both ECG and PEP, pNN50, SDNN, and RMSSD variables showed trivial ES (<0.2) and very large to nearly perfect ICC and Spearman correlation coefficient values in all time segments (>0.8). The nVLF, nLF, and nHF demonstrated a variation of ES (from trivial to small effects, 0.01-0.40), ICC (from moderate to nearly perfect, 0.39-0.96), and Spearman correlation coefficient values (from moderate to nearly perfect, 0.40-0.96). Furthermore, the Bland-Altman plots showed relatively narrow values of mean difference between the ECG and PEP after consecutive 1-minute recordings for SDNN, RMSSD, and pNN50. Acceptable limits of agreement were found after consecutive 3-minute recordings for nLF and nHF.

**Conclusions:**

Using the PEP app to facilitate a 1-minute ultra–short-term recording is suggested for time-domain HRV indices (SDNN, RMSSD, and pNN50) to interpret autonomic functions during stabilization. When using frequency-domain HRV indices (nLF and nHF) via the PEP app, a recording of at least 3 minutes is needed for accurate measurement.

## Introduction

### Background

Smartphone apps are recognized as convenient tools for our daily life activities in modern society. For health and fitness issues, there is an increasing number of smartphone users that utilize multiple free mobile phone apps to assess biosignals [1,2], psychological functions [3,4], and social behaviors in daily routines. Specific to healthy lifestyle promotion for cardiovascular functions, using a smartphone or smartwatch to monitor autonomic nervous system activities through heart rate (HR) and HR variability (HRV) is accessible and economical [5,6].

HRV is a physiological marker of cardiac autonomic responses that can be detected by recording heartbeat intervals over time. Assessment of daily HRV can provide useful information for understanding cardiac health with regards to labor force workload [7], mental conditions [8,9], and fitness status [10,11]. In general, the conventional methodology involves recording a 5-minute short-term HRV measurement, followed by a 5-minute stabilization [12].

### Ultra–Short-Term HRV Studies

Recently, ultra–short-term recordings for HRV assessment have received notable attention in cardiovascular medicine [13-15], metabolic disease [16], cognitive function [8,9], exercise testing [17-19], and sports training [11,20] studies due to the time efficiency it offers to both patients and practitioners. Ultra–short-term recording only requires R-R intervals of less than 60 seconds. Excellent limits of agreement and reproducibility of 1-minute ultra-short recordings of root mean square of successive R-R intervals (RMSSD) measurements were observed during a 5-minute stabilization period in an athletic population [11,21]. However, the methodological considerations of ultra–short-term HRV assessment have not been extensively explored in the literature. For example, a shorter time segment of less than 1 minute tended to increase measurement errors when RMSSD was log-transformed (lnRMSSD) [18].

### Study Objectives

Today, several HRV smartphone apps have been developed to evaluate autonomic health by using photoplethysmography [19,22,23]. However, the compatibility of photoplethysmographic detection is limited by physical contacts between recording locations and mobile sensors. Thus, our research group recently developed a free mobile app, Pulse Express Pro (PEP), which is compatible with wearable HR sensors and has Bluetooth functionality. The wireless app might provide an option to clients and practitioners using mobile phone–based HRV assessment. Therefore, the first aim of this study was to compare the degree of validity and reliability of ultra–short-term and short-term HRV recordings of the time-domain (standard deviation of normal R-R intervals [SDNN], RMSSD, and the proportion of NN50 divided by the total number of RR intervals [pNN50]) and frequency-domain (normalized very-low–frequency power [nVLF], normalized low-frequency power [nLF], and normalized high-frequency power [nHF]) variables with standard 5-minute assessment using a novel smartphone app, PEP. The second aim of this study was to determine the agreement of ultra–short-term and short-term HRV assessments by electrocardiogram (ECG) and PEP. We hypothesized that ultra–short-term HRV indices would show less valid and reliable measurements than that of short-term HRV indices for frequency-domain variables but not for time-domain variables.

## Methods

### Recruitment

In total, 60 healthy adults were recruited for this study (aged 22.3 [3.0] years; 168.4 [SD 8.0] cm tall; body weight: 64.2 [11.5] kg). Inclusion criteria were healthy adults aged between 20 and 30 years. Exclusion criteria included current neurological, cardiovascular, and metabolic diseases. All participants signed an informed consent form and were familiarized with experimental procedures. The participants were requested to avoid vigorous exercise 24 hours before visits and to avoid caffeine-containing substances and smoking 2 hours before the experiments. This study was approved by the Human Ethics Committee of University of Taipei (IRB-2019-005) and was conducted according to the Declaration of Helsinki and its later amendments.

Sample size was determined based on convenience and post hoc power analysis using dependent *t* tests carried out in G*Power [24]. A sample size of 60 participants demonstrated a 97% chance of obtaining a significant outcome measure at *P*<.05 with a moderate effect size (*d*=0.50).

### Experimental Procedure

**Figure 1:**
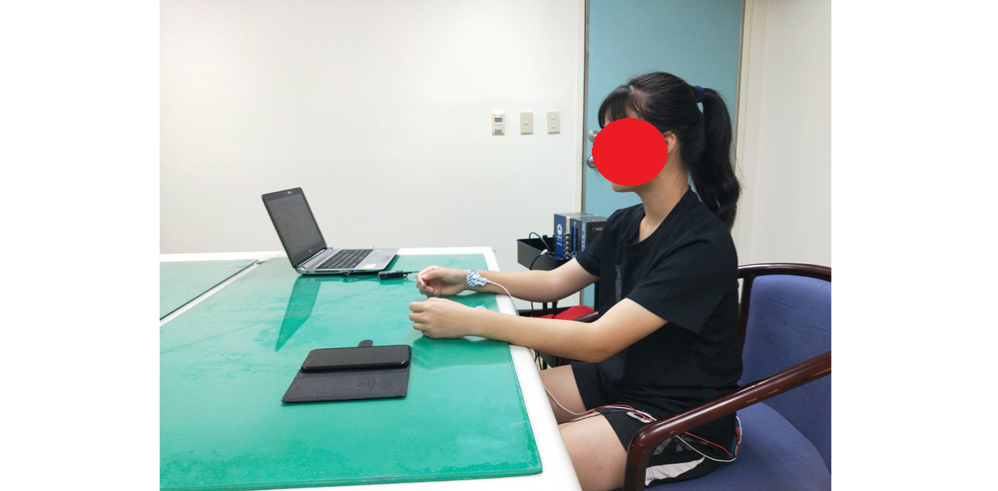
Illustration of the experimental setting and testing position.

The height and weight of each participant were measured during the first visit using a portable stadiometer (Seca 213, SECA) and electrical weight scale (Xyfwt382, Teco). At the second visit, 5-minute resting HRV data were collected in a sitting position. The ECG signals with conventional lead II arrangement were set for reference, while a portable Polar HR monitor (H7, Polar Electro) was placed on the participant’s chest for HR detection (Figure 1). A smartphone (PRA LX2, Huawei) with the PEP app [25] was used to record HRV signals via Bluetooth. The participants were instructed to breathe spontaneously during the HRV recording. The measurements were taken in a quiet and spacious room between 8 AM and 12 PM. Room temperature and humidity were controlled at around 25 °C and 70%-80%, respectively.

### HRV Recording

All participants were requested to maintain a sitting position during ECG recording. A multichannel biosignal recorder (MP160, Biopac Systems) with conventional lead II arrangement (MEC110C, Biopac Systems) was set for ECG recordings, while a telemetric HR monitoring device was used to record the resting HRV (H7, Polar Electro) via a customized smartphone app, PEP. The sampling rate of the ECG recording was set at 1000 Hz. The HRV data was exported to Google Drive and extracted to a personal laptop for data analysis. Kubios HRV Premium analysis software (Version 3.2; Kubios) was used to calculate SDNN, RMSSD, pNN50, nVLF, nLF, and nHF parameters. The SDNN, RMSSD, and pNN50 were calculated by using the standard formulas for time-domain analysis [12]. In addition, the power spectra of RR intervals were calculated by means of Fast Fourier Transformation (FFT) for frequency-domain analysis. The bands of VLF, LF, and HF ranges were set as 0-0.04 Hz, 0.04-0.15 Hz, and 0.15-0.4 Hz, respectively [12]. The normalized powers of VLF, LF, and HF were used as the autonomic indices of the participants. The formulas to calculate the normalized powers of VLF, LF, and HF bands were as follows [26-28], with nu standing for normalized unit:

nVLF[nu] = VLF (ms^2^) / total power (ms^2^) × 100 (**1**)

nLF[nu] = LF (ms^2^) / total power (ms^2^) × 100 (**2**)

nHF[nu] = HF (ms^2^) / total power (ms^2^) × 100 (**3**)

Strong artefact correction and smoothing priors set at 500Λ were used for HRV analysis to minimize the interference from Bluetooth transmission and the artefact resulting from physical contact between the chest strap and the skin [29,30]. The time segments of HRV recordings were divided into 0-1 minute, 1-2 minutes, 2-3 minutes, 3-4 minutes, and 4-5 minutes for ultra–short-term HRV recordings and 0-2 minutes, 0-3 minutes, 0-4 minutes, and 0-5 minutes (standard) for short-term HRV recordings.

### Statistical Analysis

Statistical analyses were conducted using SPSS Statistics (Version 25.0; IBM Corp) and Microsoft Excel 2013 (Microsoft Corp). Descriptive data of the measured variables are presented as median and interquartile range (25%-75%). Magnitude of difference and agreement of HRV indices in all time segments (with the 5-minute criterion as a reference) were analyzed by using the standardized differences of variables (effect size: ES), Cohen *d*. The ES was interpreted as trivial (0.0-0.2), small (0.2-0.6), moderate (0.6-1.2), large (1.2-2.0), and very large (>2.0) [31]. In terms of validity and reliability between the ECG and PEP assessments, intraclass correlation coefficients (ICC) with a two-way random model and single measure were used to determine the relative values of reliability. The ICC values were expressed as small (0.0-0.3), moderate (0.31-0.49), large (0.50-0.69), very large (0.70-0.89), and nearly perfect (0.9-1.0) [31]. The correlation coefficient between the ECG and PEP was assessed by using the Spearman rank correlation (*r*). The level of the correlation coefficients was determined as trivial (*r*<0.1), small (0.1<*r*<0.3), moderate (0.3<*r*<0.5), high (0.5<*r*<0.7), very high (0.7<*r*<0.9), nearly perfect (*r*>0.9), and perfect (*r*=1) [31]. Lastly, a Bland-Altman plot was used to evaluate the upper and lower limits of agreement among time segments of the HRV indices as determined by the ECG and PEP [32].

## Results

### Standardized Differences and Limits of Agreement

The descriptive information and standardized differences of HRV indices for all time segments of the ECG and PEP measurements are presented in Tables 1 and 2. The results showed trivial ES in all time segments of the SDNN, RMSSD, and pNN50, compared to the 0-5–minute standard measurement. In contrast, a variation of ES from trivial to small effect was found in the nVLF, nLF, and nHF variables.

**Table 1:** Median and interquartile range (25%-75%) of time-domain and frequency-domain heart rate variability parameters in different time segments of the electrocardiogram and Pulse Express PRO measurements^a^.

Device and time segment (minutes)		SDNN, ms	RMSSD, ms	pNN50, %	nVLF, nu	nLF, nu	nHF, nu
		Median (IQR)	Median (IQR)	Median (IQR)	Median (IQR)	Median (IQR)	Median (IQR)
**ECG**							
	0-5 (standard)	41.63 (30.24-53.93)	39.38 (27.73-50.56)	20.56 (6.89-35.12)	3.82 (1.80-6.85)	54.61 (41.82-65.32)	41.02 (26.17-51.47)
	0-1	37.83 (28.75-55.43)	37.76 (30.40-58.16)	19.86 (6.88-40.09)	2.92 (1.30-7.62)	47.57 (33.56-62.89)	46.02 (31.52-61.12)
	1-2	42.36 (30.27-60.03)	40.89 (26.92-55.40)	20.71 (6.47-37.20)	3.23 (1.80-7.10)	50.13 (37.36-71.45)	42.82 (23.30-59.14)
	2-3	40.08 (29.24-55.39)	38.20 (27.85-51.59)	20.32 (4.84-36.24)	5.09 (1.99-8.19)	47.92 (33.34-63.49)	45.22 (28.46-61.41)
	3-4	39.59 (30.73-54.09)	37.84 (26.30-50.93)	19.64 (4.09-32.36)	3.39 (1.20-6.57)	45.97 (31.62-65.00)	49.34 (30.12-64.34)
	4-5	39.13 (28.80-48.01)	35.27 (26.70-47.15)	15.53 (5.12-33.94)	3.77 (1.74-7.87)	49.73 (27.99-66.25)	44.25 (24.85-63.73)
	0-2	41.79 (29.81-58.74)	40.70 (29.05-56.91)	23.49 (6.37-35.78)	3.42 (1.61-6.89)	43.04 (31.61-61.63)	51.65 (26.64-62.59)
	0-3	40.87 (30.74-56.58)	40.06 (28.23-52.88)	21.84 (5.82-34.26)	3.64 (1.36-6.67)	51.41 (38.21-67.54)	42.00 (27.84-57.41)
	0-4	41.59 (30.63-57.47)	40.23 (27.66-52.04)	21.59 (7.11-35.49)	3.51 (1.91-6.62)	53.12 (43.26-66.91)	39.57 (27.73-51.41)
**PEP**							
	0-5 (standard)	41.51 (30.33-53.85)	39.09 (27.79-50.63)	20.20 (7.08-35.16)	3.90 (1.80-6.98)	54.65 (42.63-65.55)	41.92 (26.95-51.81)
	0-1	39.52 (28.65-55.03)	38.72 (31.25-57.80)	20.24 (7.91-38.26)	2.80 (1.20-7.78)	48.20 (32.93-62.48)	45.34 (32.66-60.23)
	1-2	41.80 (30.20-60.71)	40.98 (27.00-55.54)	22.84 (5.10-36.78)	3.35 (1.55-6.54)	50.16 (35.69-71.22)	42.74 (24.45-59.85)
	2-3	40.44 (30.51-55.29)	38.26 (27.54-51.65)	21.19 (4.82-36.46)	4.76 (1.91-8.75)	48.07 (33.45-62.41)	42.67 (27.55-62.10)
	3-4	39.31 (30.53-54.32)	38.73 (26.70-51.73)	20.59 (4.95-32.33)	3.43 (1.13-6.25)	46.18 (34.18-64.19)	48.33 (28.20-62.80)
	4-5	39.01 (28.76-47.35)	36.30 (26.64-47.35)	17.29 (5.11-33.81)	4.38 (1.63-7.85)	48.97 (26.83-64.78)	44.56 (24.59-64.24)
	0-2	42.90 (29.78-59.09)	40.90 (29.25-57.07)	24.67 (6.62-37.26)	3.46 (1.71-7.22)	42.11 (31.50-59.15)	52.88 (26.82-64.49)
	0-3	41.62 (31.14-56.75)	39.99 (28.09-53.32)	22.13 (5.89-35.72)	3.84 (1.33-6.55)	51.19 (38.00-67.06)	41.64 (28.85-57.86)
	0-4	41.43 (30.74-57.72)	40.18 (27.79-52.08)	21.78 (6.92-35.22)	3.46 (1.92-6.70)	53.55 (42.65-67.67)	39.36 (29.08-51.90)

^a^ECG: electrocardiogram; ms: milliseconds; nHF: normalized high-frequency power; nLF: normalized low-frequency power; nu: normalized unit; nVLF: normalized very-low–frequency power; PEP: Pulse Express PRO; pNN50: proportion of NN50 divided by the total number of RR intervals; RMSSD: root mean square of successive R-R intervals; SDNN: standard deviation of normal R-R intervals.

**Table 2:** Standardized differences (95% CI) of time-domain and frequency-domain heart rate variability parameters in different time segments of the electrocardiogram and Pulse Express PRO measurements compared with the 0-5–minute standard^a^.

Device and time segment (minutes)		SDNN (95% CI)		RMSSD (95% CI)		pNN50 (95% CI)		nVLF (95% CI)		nLF (95% CI)		nHF (95% CI)	
**ECG**													
	0-1		0.01 (–0.35 to 0.36)		–0.07 (–0.43 to 0.29)		–0.10 (–0.46 to 0.26)		–0.03 (–0.39 to 0.33)		0.40 (0.04 to 0.76)		–0.38 (–0.74 to –0.02)
	1-2		–0.12 (–0.48 to 0.24)		–0.04 (–0.39 to 0.32)		–0.09 (–0.44 to 0.27)		0.05 (–0.31 to 0.41)		0.08 (–0.28 to 0.44)		–0.09 (–0.45 to 0.27)
	2-3		0.05 (–0.31 to 0.41)		0.05 (–0.31 to 0.41)		0.01 (–0.34 to 0.37)		–0.01 (–0.61 to 0.11)		0.34 (–0.02 to 0.71)		–0.26 (–0.62 to 0.10)
	3-4		0.05 (–0.31 to 0.40)		0.05 (–0.31 to 0.41)		0.04 (–0.32 to 0.40)		–0.04 (–0.40 to 0.32)		0.29 (–0.07 to 0.65)		–0.27 (–0.63 to 0.08)
	4-5		0.12 (–0.23 to 0.48)		0.08 (–0.28 to 0.44)		0.11 (–0.24 to 0.47)		–0.20 (–0.56 to 0.16)		0.25 (–0.11 to 0.61)		–0.20 (–0.56 to 0.16)
	0-2		–0.07 (–0.43 to 0.29)		0.06 (–0.41 to 0.30)		–0.09 (–0.45 to 0.26)		–0.02 (–0.37 to 0.34)		0.35 (0.00 to 0.72)		–0.34 (–0.70 to 0.02)
	0-3		0.04 (–0.40 to 0.32)		–0.03 (–0.38 to 0.33)		–0.06 (–0.41 to 0.30)		0.14 (–0.22 to 0.49)		0.08 (–0.28 to 0.44)		–0.11 (–0.46 to 0.25)
	0-4		0.03 (–0.38 to 0.33)		–0.01 (–0.37 to 0.34)		–0.03 (–0.39 to 0.33)		0.06 (–0.30 to 0.42)		0.01 (–0.35 to 0.37)		–0.02 (–0.38 to 0.34)
**PEP**													
	0-1		0.01 (–0.35 to 0.37)		–0.07 (–0.43 to 0.29)		–0.10 (–0.46 to 0.26)		0.01 (–0.35 to 0.36)		0.38 (0.02 to 0.74)		–0.37 (–0.73 to –0.01)
	1-2		–0.12 (–0.48 to 0.24)		–0.03 (–0.39 to 0.32)		–0.08 (–0.44 to 0.27)		0.05 (–0.31 to 0.41)		0.09 (–0.26 to 0.45)		–0.10 (–0.46 to 0.26)
	2-3		0.05 (–0.31 to 0.41)		0.06 (–0.30 to 0.42)		0.03 (–0.33 to 0.39)		–0.25 (–0.61 to 0.10)		0.33 (–0.03 to 0.69)		–0.24 (–0.60 to 0.11)
	3-4		0.04 (–0.31 to 0.40)		0.04 (–0.31 to 0.40)		0.04 (–0.32 to 0.40)		–0.07 (–0.43 to 0.29)		0.25 (–0.11 to 0.61)		–0.23 (–0.59 to 0.13)
	4-5		0.12 (–0.24 to 0.48)		0.08 (–0.28 to 0.44)		0.10 (–0.26 to 0.46)		–0.23 (–0.59 to 0.13)		0.30 (–0.06 to 0.66)		–0.23 (–0.59 to 0.13)
	0-2		–0.06 (–0.42 to 0.29)		–0.06 (–0.41 to 0.30)		–0.09 (–0.45 to 0.27)		–0.03 (–0.39 to 0.33)		0.38 (0.03 to 0.75)		–0.36 (–0.73 to 0.00)
	0-3		–0.03 (–0.39 to 0.33)		–0.02 (–0.38 to 0.33)		–0.05 (–0.41 to 0.31)		0.13 (–0.23 to 0.49)		0.10 (–0.26 to 0.45)		–0.12 (–0.48 to 0.24)
	0-4		–0.02 (–0.38 to 0.34)		–0.01 (–0.37 to 0.34)		–0.03 (–0.38 to 0.33)		0.06 (–0.30 to 0.42)		0.02 (–0.34 to 0.37)		–0.03 (–0.39 to 0.33)

^a^ECG: electrocardiogram; nHF: normalized high-frequency power; nLF: normalized low-frequency power; nVLF: normalized very-low–frequency power; PEP: Pulse Express PRO; pNN50: proportion of NN50 divided by the total number of RR intervals; RMSSD: root mean square of successive R-R intervals; SDNN: standard deviation of normal R-R intervals.

In Table 3, the Bland-Altman analysis demonstrated relatively small bias in all comparisons of the SDNN, RMSSD, pNN50, and nVLF. In contrast, a relatively small bias in the nLF and nHF variables occurred during short-term recordings of 0-3 minutes and 0-4 minutes.

**Table 3:** Limits of agreement (± 1.96*SD) of time-domain and frequency-domain heart rate variability parameters in different time segments of the electrocardiogram and Pulse Express PRO measurements compared with the 0-5–minute standard^a^.

Device and time segment (minutes)		SDNN (± 1.96*SD)		RMSSD (± 1.96*SD)		pNN50 (± 1.96*SD)		nVLF (± 1.96*SD)		nLF (± 1.96*SD)		nHF (± 1.96*SD)	
**ECG**													
	0-1		0.01 (–14.23 to 14.43)		–1.40 (–13.48 to 10.69)		–1.93 (–17.10 to 13.24)		–0.15 (–9.88 to 9.58)		7.75 (–25.66 to 41.16)		–7.63 (–40.54 to 25.29)
	1-2		–2.31 (–18.53 to 13.96)		–0.72 (–11.84 to 10.40)		–1.61 (–15.16 to 11.94)		0.21 (–8.49 to 8.92)		1.59 (–30.41 to 33.59)		–1.80 (–32.81 to 29.21)
	2-3		0.83 (–12.63 to 14.30)		0.93 (–8.46 to 10.32)		0.26 (–9.93 to 10.44)		–1.30 (–8.59 to 6.00)		6.63 (–21.17 to 34.44)		–5.32 (–33.83 to 23.19)
	3-4		0.83 (–14.72 to 16.37)		0.92 (–11.86 to 13.71)		0.70 (–11.20 to 12.60)		–0.20 (–10.39 to 10.00)		5.73 (–25.74 to 37.20)		–5.54 (–37.56 to 26.49)
	4-5		2.18 (–13.70 to 18.05)		1.64 (–13.16 to 16.44)		2.04 (–11.47 to 15.56)		–1.00 (–11.78 to 9.82)		5.08 (–34.82 to 44.98)		–4.12 (–42.78 to 34.54)
	0-2		–1.24 (–13.01 to 10.53)		–1.14 (–10.90 to 8.62)		–1.73 (–13.96 to 10.51)		–0.06 (–12.11 to 12.00)		7.07 (–26.77 to 40.92)		–7.03 (–40.37 to 26.32)
	0-3		0.66 (–7.35 to 8.66)		0.52 (–6.28 to 7.32)		–1.01 (–6.62 to 8.64)		–0.52 (–6.85 to 5.82)		–1.50 (–24.85 to 21.85)		2.03 (–20.03 to 24.09)
	0-4		–0.44 (–4.14 to 3.26)		–0.28 (–3.85 to 3.30)		–0.54 (–3.91 to 2.84)		0.24 (–2.78 to 3.26)		–0.15 (–10.98 to 11.28)		–0.38 (–11.00 to 10.23)
**PEP**													
	0-1		0.20 (–13.41 to 13.81)		–1.35 (–13.06 to 10.36)		–1.86 (–16.16 to 12.43)		0.03 (–9.77 to 9.82)		7.32 (–26.30 to 40.94)		–7.37 (–40.44 to 25.70)
	1-2		–2.24 (–22.67 to 18.91)		–0.70 (–21.94 to 20.54)		–1.60 (–24.24 to 21.04)		0.20 (–8.56 to 8.96)		1.84 (–32.13 to 35.80)		–2.04 (–36.20 to 32.12)
	2-3		0.89 (–21.01 to 22.79)		1.18 (–18.49 to 20.84)		0.53 (–17.35 to 18.41)		–1.40 (–9.61 to 6.82)		6.35 (–22.36 to 35.06)		–4.94 (–34.00 to 24.11)
	3-4		0.80 (–19.94 to 21.55)		0.88 (–18.35 to 20.10)		0.71 (–15.93 to 17.35)		–0.31 (–10.53 to 9.92)		4.93 (–26.76 to 36.62)		–4.61 (–37.63 to 28.42)
	4-5		2.06 (–16.98 to 21.09)		1.58 (–20.36 to 23.52)		1.75 (–18.13 to 21.63)		–0.63 (–10.11 to 8.84)		5.87 (–31.94 to 43.69)		–4.74 (–41.70 to 32.23)
	0-2		–1.16 (–12.68 to 11.81)		–1.12 (–10.86 to 8.62)		–1.69 (–13.91 to 10.52)		–0.99 (–10.56 to 8.57)		7.62 (–25.58 to 40.83)		–7.51 (–40.22 to 25.19)
	0-3		–0.57 (–8.65 to 7.52)		–0.46 (–7.25 to 6.33)		–0.91 (–8.66 to 6.85)		–0.22 (–6.23 to 5.79)		1.78 (–21.56 to 25.12)		–2.29 (–24.52 to 19.94)
	0-4		–0.38 (–4.28 to 3.52)		–0.25 (–3.74 to 3.24)		–0.45 (–3.87 to 2.97)		–0.08 (–2.41 to 2.24)		0.28 (–10.83 to 11.38)		0.51 (–11.16 to 10.14)

^a^ECG: electrocardiogram; nHF: normalized high-frequency power; nLF: normalized low-frequency power; nVLF: normalized very-low–frequency power; PEP: Pulse Express PRO; pNN50: proportion of NN50 divided by the total number of RR intervals; RMSSD: root mean square of successive R-R intervals; SDNN: standard deviation of normal R-R intervals.

### Intraclass Correlation Coefficients

**Figure 2:**
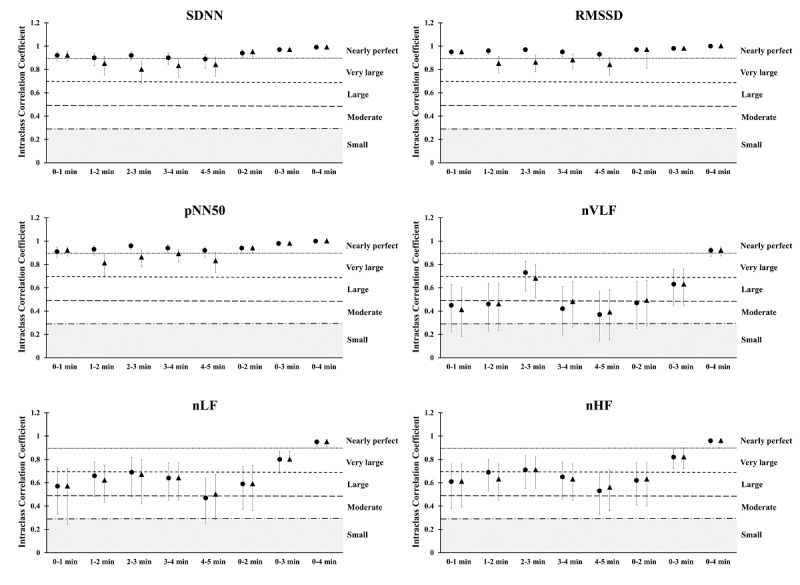
Intraclass correlation coefficients between the ECG and Pulse Express PRO measurements in ultra–short-term and short-term heart rate variability in time-domain and frequency-domain analyses. The grey area indicates low reliability. The black circle indicates the ECG recordings, while the black triangle indicates the Pulse Express PRO recordings. ECG: electrocardiogram.

The results demonstrated similar outcomes for ICC values for the ECG and PEP measurements. The SDNN, RMSSD, and pNN50 ICC values were nearly perfect in all ultra–short-term and short-term records compared to the 0-5–minute standard ECG measurement (from very large to nearly perfect, 0.89-1.0). Furthermore, the time-domain variables of PEP were very large to nearly perfect for ultra–short-term recordings, except the 0-1–minute time segment (0.81-0.94). In terms of frequency-domain analysis, nearly perfect ICC values were found in the 0-4–minute time segment of the nVLF, nLF, and nHF (0.92-0.96). Very large ICC values were found in the 0-3–minute time segments for nLF and nHF (0.80-0.82). A broad range of ICC values was identified among the other comparisons (from moderate to very large, 0.37-0.71; Figure 2).

### Correlation Coefficient

**Figure 3:**
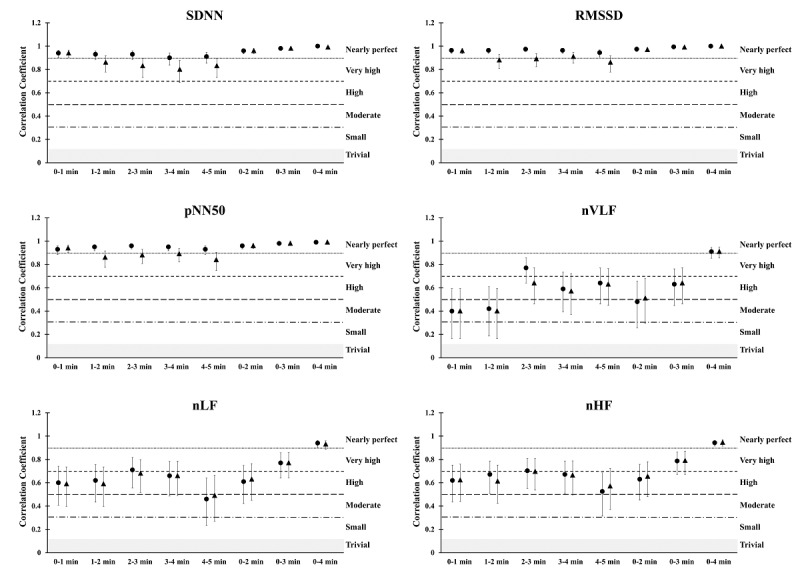
Spearmen correlation coefficients between the ECG and Pulse Express PRO measurements in ultra–short-term and short-term heart rate variability in time-domain and frequency-domain analyses. The grey area indicates trivial correlation coefficient values. The black circle indicates the ECG recordings, while the black triangle indicates the Pulse Express PRO recordings. ECG: electrocardiogram.

Compared to the 0-5–minute standard measurement, the Spearman correlation coefficients were nearly perfect for the SDNN, RMSSD, and pNN50 variables in all time segments for the ECG measurements (0.90-1.0). Furthermore, the correlation coefficients were very large for the time-domain variables for ultra–short-term recordings using PEP (0.80-1.0), except for nearly perfect values for the 0-1–minute time segment. For frequency-domain analysis, a nearly perfect correlation coefficient was only found for 0-4–minute recordings (0.91-0.96). Furthermore, a very large correlation coefficient was found in the nLF and nHF 0-3–minute recordings (0.77-0.81). In contrast, a wide range of values was identified among the other comparisons (from moderate to very large, 0.40-0.77; Figure 3).

### Bland-Altman Plots Comparing ECG and PEP Measurements

**Figure 4:**
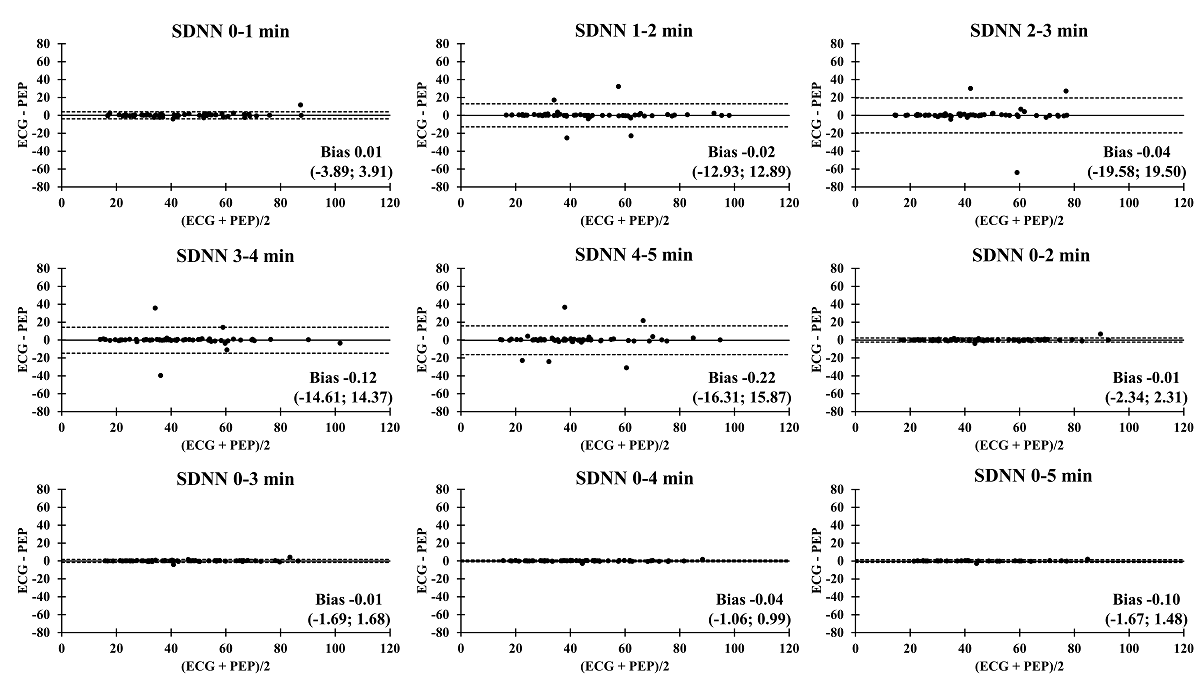
Bland-Altman analysis comparing the ECG and Pulse Express PRO measurements in ultra–short-term and short-term recordings of standard deviation of normal R-R intervals (SDNN). The solid line represents the mean difference and the upper and lower dashed lines represent the upper and lower limits of agreement (± 1.96*SD). ECG: electrocardiogram; PEP: Pulse Express PRO.

**Figure 5:**
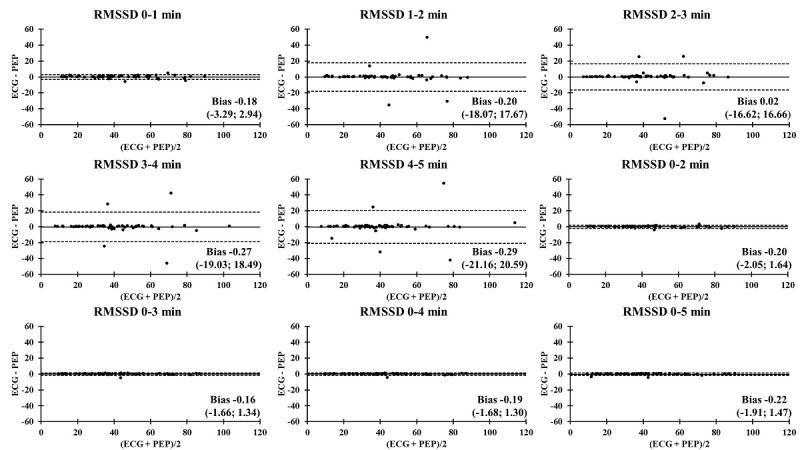
Bland-Altman analysis comparing the ECG and Pulse Express PRO measurements in ultra–short-term and short-term recordings of root mean square of successive R-R interval (RMSSD). The solid line represents the mean difference and the upper and lower dashed lines represent the upper and lower limits of agreement (± 1.96*SD). ECG: electrocardiogram; PEP: Pulse Express PRO.

**Figure 6:**
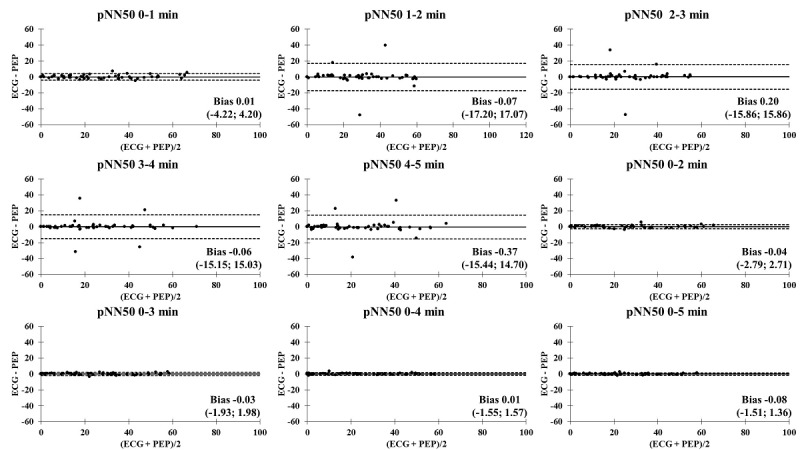
Bland-Altman analysis comparing the ECG and Pulse Express PRO measurements in ultra–short-term and short-term recordings of proportion of NN50 divided by the total number of RR intervals (pNN50). The solid line represents the mean difference and the upper and lower dashed lines represent the upper and lower limits of agreement (± 1.96*SD). ECG: electrocardiogram; PEP: Pulse Express PRO.

**Figure 7:**
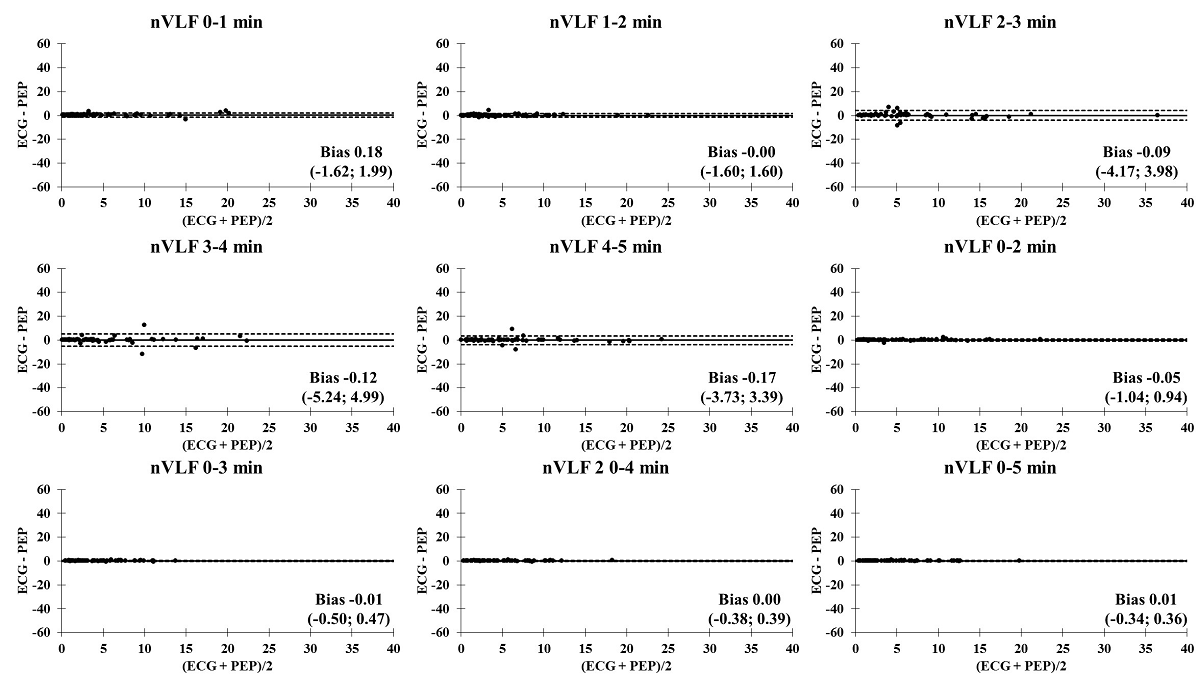
Bland-Altman analysis comparing the ECG and Pulse Express PRO measurements in ultra–short-term and short-term recordings of normalized very low frequency power (nVLF). The solid line represents the mean difference and the upper and lower dashed lines represent the upper and lower limits of agreement (± 1.96*SD). ECG: electrocardiogram; PEP: Pulse Express PRO.

**Figure 8:**
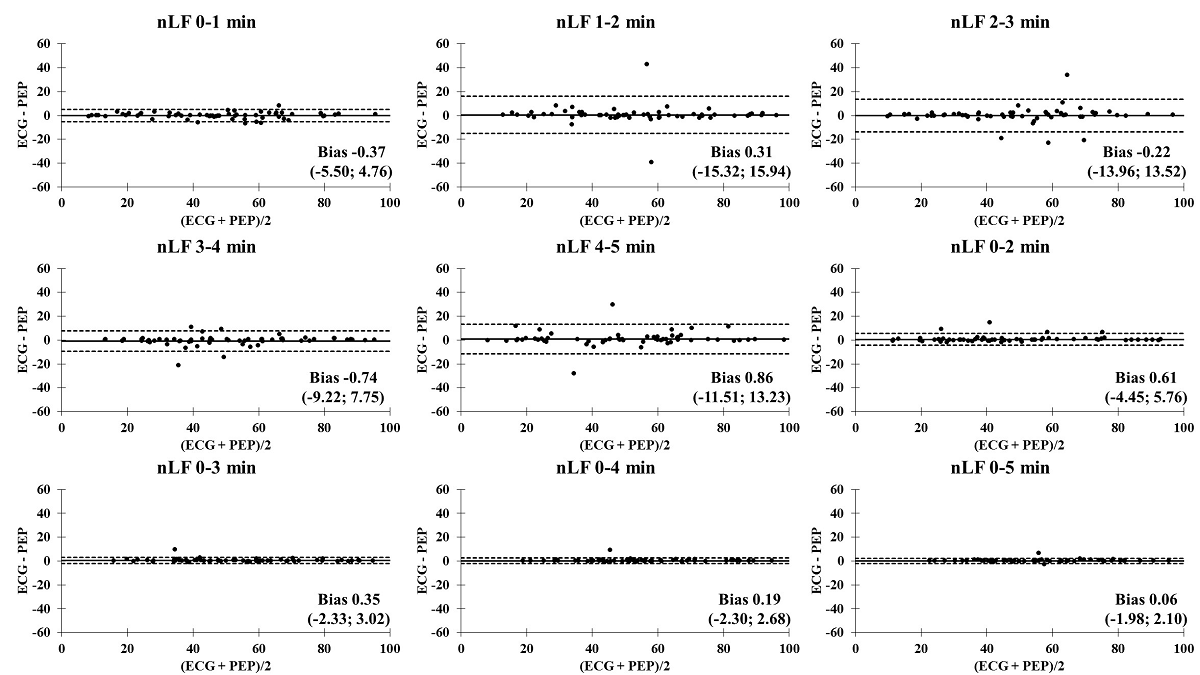
Bland-Altman analysis comparing the ECG and Pulse Express PRO measurements in ultra–short-term and short-term recordings of normalized low frequency power (nLF). The solid line represents the mean difference and the upper and lower dashed lines represent the upper and lower limits of agreement (± 1.96*SD). ECG: electrocardiogram; PEP: Pulse Express PRO.

**Figure 9:**
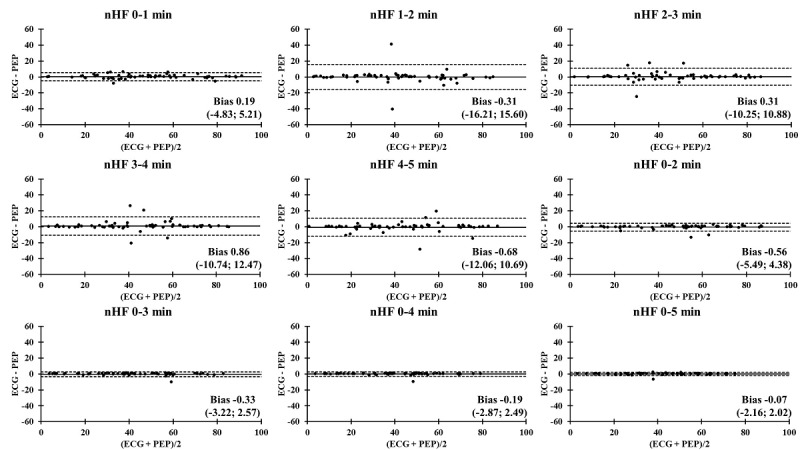
Bland-Altman analysis comparing the ECG and Pulse Express PRO measurements in ultra–short-term and short-term recordings of normalized high frequency power (nHF). The solid line represents the mean difference and the upper and lower dashed lines represent the upper and lower limits of agreement (± 1.96*SD). ECG: electrocardiogram; PEP: Pulse Express PRO.

The Bland-Altman plots comparing the ECG and PEP measurements showed relatively narrow values of mean difference in all time segments (Figures 4-9). In addition, the Bland-Altman analysis found a narrow standard deviation for consecutive 2-minute recordings for SDNN, RMSSD, pNN50, and nVLF. In addition, acceptable limits of agreement were found after consecutive 3-minute recordings for nLF and nHF.

## Discussion

### Principal Results

This study is the first to report the validity and reliability of ultra–short-term and short-term HRV via a novel smartphone app, and to compare the app with the standard ECG assessment. The limits of agreement of HRV assessments between the ECG and PEP were compared to evaluate the accuracy of measurements. The primary finding in the present study was that SDNN, RMSSD, and pNN50 parameters had very large to nearly perfect ICC and Spearman correlation coefficients in all time segments. Additionally, a large variation in ICC and Spearman correlation coefficients was found in time segments under 2 minutes for the nVLF, nLF, and nHF parameters. The 3-minute and 4-minute nLF and nHF HRV recordings showed excellent validity and reliability and could be considered a surrogate of the standard 5-minute recording. Furthermore, with the ECG signal as a reference, the accuracy of PEP HRV recordings can be found with consecutive 1-minute recordings in the time-domain analysis (SDNN, RMSSD, and pNN50). Lastly, for the frequency-domain analysis (nLF and nHF), a recording of at least 3 minutes is required for accurate and valid PEP HRV assessment.

### Time-Domain Analysis

Based on our observations, a 1-minute ultra–short-term HRV recording for the time-domain analysis revealed valid and reliable HRV features (with the 5-minute criterion as reference), despite an initial 5-minute stabilization. This indicates that the PEP app is a convenient surrogate for taking HRV measurements. It is suggested that the RMSSD is independent of respiratory sinus arrhythmia and is associated with high-frequency changes of HR modulation in response to respiratory patterns due to its strength of mathematical calculation [33]. The RMSSD has been widely accepted to evaluate cardiac-related parasympathetic activation [8,11,13,18,19,34]. Additionally, the RMSSD is recognized as a sensitive parameter to detect autonomic adaptations in response to mental stress [8,35,36] and psychophysiological strain after exercise as well as recovery status during the training period [10,37]. Long-term monitoring of resting HRV can provide valuable information to identify the chronological development of vagal-related changes related to psychometric status during sports training [38]. As demonstrated by our findings, PEP could be considered an alternative tool for short-term HRV measurements.

It is arguable that the PEP presented valid and reliable measurements in SDNN accompanied by RMSSD and pNN50 for any HRV epoch. It seems that SDNN and pNN50 are good options to integrate time-domain HRV indices. However, as the accuracy of ultra–short-term measurements of SDNN may be influenced by psychological conditions (ie, being under mental stress) [8,13], using the PEP app to facilitate 1-minute ultra–short-term HRV recordings in a quiet and relaxed manner is documented in this study.

### Frequency-Domain Analysis

It is important to note that nVLF, nLF, and nHF showed trivial or small differences in association with a large variation in ICC values, correlation coefficients, and bias across all time segments compared to the standard 0-5–minute criterion. The poor validity and reliability of nVLF, nLF, and nHF in shortened epochs could be related to interindividual variations in breathing rates during measurements. Interindividual variations in breathing patterns could increase the risk of increasing HR oscillations in different time segments. Respiratory rhythm is thought of as an essential way to record frequency-domain variables such as LF and HF due to oscillations in HR responses [39]. However, breath control during resting HRV measurement does not increase accuracy and reliability during short-term recordings of frequency-domain analysis [9]. Control of respiratory frequency is not common in the general population (ie, people without appropriate respiratory training). Thus, we did not apply this instruction due to limited popularity of use.

Our findings suggest using consecutive HRV recordings of at least 3 minutes when the PEP app is used to monitor frequency-domain variables. In contrast, the minimum time requirement for HF and LF recordings has been suggested as 1 and 2 minutes, respectively [13,40]. Castaldo et al [8] showed accurate frequency-domain measurements in 1 minute for HF and 2 minutes for LF recordings after university examinations. The inconsistent findings of this study might be related to the different spectral analysis computational methods (spectrum resolution: FFT versus autoregressive) and the stabilization period prior to the HRV measurement.

### Bland-Altman Analysis Comparing ECG and PEP Measurements

In an attempt to identify the agreement of biosignal measurements between the ECG and PEP, a Bland-Altman analysis was performed to compare the limit of agreement of ultra–short-term and short-term HRV recordings of the SDNN, RMSSD, pNN50, nVLF, nLF, and nHF. It is interesting to note that the PEP HRV recordings showed similar outcomes for the SDNN, RMSSD, pNN50, nVLF, nLF, and nHF measurements for all time segments, as compared to conventional lead II ECG recordings. This study revealed the accuracy and acceptance of PEP HRV recordings after consecutive 1-minute recordings in the time-domain analysis. In contrast, the degree of agreement between the ECG and PEP was relatively low for the first 3-minute assessment when frequency-domain analysis was computed. One possible explanation for less accurate measurements of frequency-domain HRV variables with shorter duration recordings may be the lack of a detrending method for processing spectral signals in the PEP app [41]. Another factor that influences measurement accuracy is related to obtaining an adequate amount of data throughout the entire measurement [42]. Lastly, acute adaptation to postural changes from standing to sitting (orthostatic stress) might be a potential mechanism to attenuate valid and reliable measurements of nLF and nHF during the 3-minute stabilization period [43,44]. Nevertheless, the PEP app is an acceptable option for HRV data collection due to its convenience and reproducibility compared to the ECG assessment.

### Limitations

The first limitation of this study is that a telemetric HR sensor and a chest strap were required to detect HR responses during the PEP measurement, and that these accessories may not be commonly owned by the general population. In addition, the recording position and the HR chest strap might not be comfortable for specific populations (ie, senior adults) and clinical settings. Despite the abovementioned limitations, this is a novel study that reports the validity and accuracy of the PEP app for short-term HRV recordings.

### Functional Implication

Time management is critical for professionals, including clinical practitioners and strength and conditioning coaches of elite sports teams. The PEP app is compatible with the Android operating system and can be used on low-cost smartphones. As growing numbers of studies focus on the methodological issues related to utilizing ultra–short-term HRV recordings, the number of nonprofessionals using this free mobile app can easily be increased. We suggest that future studies should examine the use of PEP HRV assessments in the context of multidisciplinary approaches (eg, longitudinal applications in monitoring training loads, daily evaluations during competitions, and clinical evaluation).

The accuracy and reliability of the LF and HF measurements are critical to interpreting the shift of sympathovagal activities [33,45]. Excellent validity and reliability of the SDNN and RMSSD during ultra–short-term recordings indicated that the SDNN:RMSSD ratio might be appropriate to use in the first minute of PEP recording. The SDNN:RMSSD ratio is a sensitive HRV parameter that indicates autonomic adaptation in response to pathological conditions [45] and acute exercise [46]. Taking into consideration time efficiency and cross-battery assessment, our findings support the use of the SDNN:RMSSD ratio as a surrogate for the LF:HF ratio to estimate sympathovagal balance via a smartphone app.

### Conclusions

In conclusion, the PEP smartphone app provides reliable and valid HRV data. It is appropriate to use the PEP app to facilitate 1-minute ultra–short-term HRV recordings during stabilization to save time when the time-domain analysis is used. Caution should be taken when the frequency-domain analysis is implemented for the interpretation of cardiac autonomic modulation. Consecutive recordings of at least 3 minutes during stabilization are suggested for accurate measurement of frequency-domain nLF and nHF indices. The use of the PEP smartphone app for ultra–short-term and short-term HRV recordings is recommended as an easy and user-friendly tool to monitor cardiac autonomic health in people with various lifestyles.

## Abbreviations

ECG: electrocardiogram
ES: effect size
FFT: Fast Fourier Transformation
HR: heart rate
HRV: heart rate variability
ICC: intraclass correlation coefficient
lnRMSSD: natural logarithm of root mean square differences between adjacent normal R-R intervals
nHF: normalized high frequency power
nLF: normalized low frequency power
nVLF: normalized very low frequency power
PEP: Pulse Express PRO
pNN50: the proportion of NN50 divided by the total number of RR intervals
RMSSD: root mean square differences between adjacent normal R-R intervals
SDNN: standard deviation of normal R-R intervals
